# Bacteriocins in the Era of Antibiotic Resistance: Rising to the Challenge

**DOI:** 10.3390/pharmaceutics13020196

**Published:** 2021-02-02

**Authors:** Gratiela Gradisteanu Pircalabioru, Laura Ioana Popa, Luminita Marutescu, Irina Gheorghe, Marcela Popa, Ilda Czobor Barbu, Rodica Cristescu, Mariana-Carmen Chifiriuc

**Affiliations:** 1 Research Institute of the University of Bucharest, Earth, Environmental and Life Sciences, Section-ICUB, 050663 Bucharest, Romania; gratiela.gradisteanu@icub.unibuc.ro (G.G.P.); lpbio_laura@yahoo.com (L.I.P.); iryna_84@yahoo.com (I.G.); bmarcelica@yahoo.com (M.P.); ilda.czobor@yahoo.com (I.C.B.); carmen.chifiriuc@gmail.com (M.-C.C.); 2 Lasers Department, National Institute for Lasers, Plasma and Radiation Physics, P.O. Box MG-36, RO-077125 Magurele, Romania; 3 Academy of Romanian Scientists, 050045 Bucharest, Romania

**Keywords:** bacteriocins, nisin, antibiotic resistance

## Abstract

Decades of antibiotic misuse in clinical settings, animal feed, and within the food industry have led to a concerning rise in antibiotic-resistant bacteria. Every year, antimicrobial-resistant infections cause 700,000 deaths, with 10 million casualties expected by 2050, if this trend continues. Hence, innovative solutions are imperative to curb antibiotic resistance. Bacteria produce a potent arsenal of drugs with remarkable diversity that are all distinct from those of current antibiotics. Bacteriocins are potent small antimicrobial peptides synthetized by certain bacteria that may be appointed as alternatives to traditional antibiotics. These molecules are strategically employed by commensals, mostly Firmicutes, to colonize and persist in the human gut. Bacteriocins form channels in the target cell membrane, leading to leakage of low-molecular-weight, causing the disruption of the proton motive force. The objective of this review was to list and discuss the potential of bacteriocins as antimicrobial therapeutics for infections produced mainly by resistant pathogens.

## 1. Introduction

Factors such as use of multiple broad-spectrum agents and globalization, as well as the excessive use of antibiotics both in clinical settings and agriculture, have potentiated the emergence of pathogens resistant to single and, subsequently, multiple antibiotics, making it harder to treat common infectious diseases, hence leading to prolonged illness with increased rates of morbidity and mortality [1].

**Figure 1:**
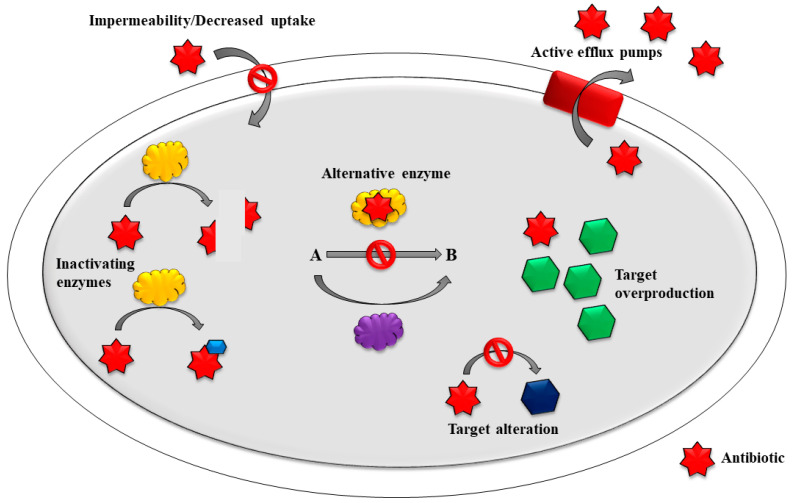
Mechanisms of antibiotic resistance; CC-BY-4.0 license.

The mechanisms of drug resistance are grouped into three main categories: (1) drug inactivation by irreversible enzymatic cleavage/modification; (2) target modification at the site of antibiotic binding; and (3) reduced drug accumulation due to low permeability or to increased drug efflux [2] (Figure 1).

Antibiotic-resistant microorganisms are classified by the CDC (Centers for Disease Control and Prevention) 2019 AR (antibiotic resistance) Threats Report, depending on the emergency and severity of the required actions, as urgent threats (carbapenem-resistant *Acinetobacter*, *carbapenem-resistant Enterobacteriaceae, Clostridium difficile, Candida auris,* drug-resistant *Neisseria gonorrhoeae*), serious threats (drug-resistant tuberculosis, multidrug-resistant *Pseudomonas aeruginosa*, drug ESBL (Extended-spectrum beta-lactamases)-producing Enterobacteriaceae, drug-resistant *Salmonella* serotype Typhi, drug-resistant *Campylobacter*, drug-resistant *Shigella*, drug-resistant nontyphoidal *Salmonella*, drug-resistant *Candida*, methicillin-resistant *Staphylococcus aureus*, vancomycin-resistant enterococci, drug -resistant *Streptococcus pneumoniae*), concerning threats (clindamycin-resistant group B, *Streptococcus* erythromycin-resistant group A *Streptococcus*), and watch list (drug-resistant *Bordetella pertussis*, drug-resistant *Mycoplasma genitalium*, azole-resistant *Aspergillus fumigatus*) [3,4].

Some of the most dangerous microbial threats in terms of resistance are united under the acronym “ESKAPE” (*Enterococcus faecium*, *Staphylococcus aureus*, *Klebsiella pneumoniae*, *Acinetobacter baumannii*, *Pseudomonas aeruginosa*, and *Enterobacter* species), presently becoming ESCAPE (*Enterococcus faecium*, *Staphylococcus aureus*, *Clostridium difficile,* *Acinetobacter baumannii*, *Pseudomonas aeruginosa*, and *Enterobacteriaceae*). These clinically important pathogens often harbor mobile genetic elements, facilitating the spread of resistant organisms as well as the ability to develop biofilms on viable host tissues or inert substrata [5]. As their acronym suggests, these pathogens are able to “escape” the biocidal action of antimicrobial agents and they are major culprits of nosocomial infections linked to the highest risk of mortality and elevated health care costs [6,7]. ESKAPE pathogens are listed by the World Health Organization (WHO) among the bacteria against which novel antimicrobials are urgently needed. The urgency to develop new antibiotics was classified into medium, high, and critical priority. The critical priority list is comprised of extended spectrum β-lactamase (ESBL) or carbapenem-resistant *K. pneumoniae* and *Enterobacter* spp., while carbapenem-resistant *A. baumannii* and *P. aeruginosa* are labeled as critical priority pathogens. On the other hand, vancomycin-resistant *E. faecium (VRE)* and methicillin- and vancomycin-resistant *S. aureus* (MRSA and VRSA) are considered high priority pathogens [8].

At the same time, there is a scarcity of new families of drugs that alleviate the resistance to current antibiotics, mostly due to the risks and high production costs that are related to the development of such products.

Worryingly, it is estimated that by 2050 there will be no efficient antibiotic available to treat infections, if no new drugs are produced [9]. At the moment, infections triggered by Gram-negative bacteria are of main concern. Gram-negative pathogens (*Pseudomonas aeruginosa, Klebsiella pneumoniae, Escherichia coli, Acinetobacter baumannii*) have an impermeable outer membrane that hinders the entrance of many classes of antibiotics. This subsequently leaves narrow therapeutic options, making them gradually less successful, while resistance spreads and patient outcomes are increasingly poor [10].

Therefore, alternative methods to combat antibiotic-resistant pathogens are urgently needed. Among the alternative methods that have been investigated, a promising lead is offered by antimicrobial peptides from a variety of sources, including bacteriocins. Bacteriocins can be defined as biologically active peptides harboring a bactericidal mode of action, which, although variable among various bacteriocin types, are distinct from those of current chemotherapeutic agents.

Compelling features of bacteriocins that underscore their viability as alternative to conventional antibiotics include: (1) single-strike kinetics, a single molecule of bacteriocin invading the target cell being able to kill it; (2) biological activity against all known human and animal pathogens and efficient in a wide spectrum of infections: cutaneous, throat, bladder, bloodstream and gut; (3) rapid killing/inhibiting mechanisms against both metabolically latent and active cells; (4) MIC (Minimum inhibitory concentration) values comparable with those of conventional antibiotics; (5) stable antimicrobial activity under a broad range of ecological factors (temperature and pH); and (6) selection for mutations associated with resistance is not occurring in several species at the same time, as in the case of broad-spectrum antibiotics [11]. The diverse structure of bacteriocins and the high level of post-translational modifications (cyclization, disulfide bridges, and nonconventional amino acids) make them typically less labile than antibiotics, hence, they can support high temperatures and extreme pH.

The objective of this review was to update and discuss the potential of bacteriocins as promising therapeutics against the most threatening resistant microorganisms.

## 2. Bacteriocins: General Features

A widespread antimicrobial strategy employed by the innate immune system of several forms of life, from insects to plants, reptiles, and humans, is represented by the production of antimicrobial peptides (AMPs). Bacteriocins are small AMPs of 30–60 amino acids produced by Gram-positive and Gram-negative bacteria, ribosomally synthesized, and very heterogeneous regarding their size, structure, mechanisms of action, spectrum of activity, biochemical properties, and target cell receptors. It is considered that the majority of bacteria (mostly Gram-positive) and archaea generate at least one antimicrobial peptide for self-preservation and competitive advantages in their ecological niche [12].

A study by Drissi et al. (2015) suggests that bacteriocins are widespread across the human gastrointestinal tract with 317 genomes encoding putative bacteriocins of classes I (44%), II (38.6%), and III (17.3%). Out of the 317 putative bacteriocins, the majority (175) were members of the Firmicutes phyla (which include Lactobacilli), 79 were Proteobacteria, and the rest Actinobacteria (25) and Bacteroidetes (34) [13]. The relatively high number of putative bacteriocins belonging to Proteobacteria may justify why they are highly virulent and persistent. These putative bacteriocins produced by the gut microbiota contain less leucine, aspartic acid, glutamine, and arginine, but more lysine and methionine and are smaller in size compared to other bacteriocins. Moreover, Drissi et al. (2015) suggested that bacteriocins in the gut microbiota may exhibit low antimicrobial activity and, therefore, not hold a drastic impact on the microbiota [13]. The main bacteriocin-producing Gram-negative bacteria are the *Enterobacteriaceae*, especially *E. coli* strains, several isolates being demonstrated to produce such antagonistic compounds as response to stress conditions.

Due to their antimicrobial properties, bacteriocins have wide applications including as additives to packaging materials for pharmaceuticals, cosmetic products, and foods, extending their shelf life and expiration date [14,15].

The classification schemes for bacteriocins are constantly changing in order to accommodate their increased diversity and complexity. Based on their origin and intrinsic function, physicochemical properties, molecular weight, and amino acid sequence, bacteriocins are divided into several classes (Table 1).

Class I bacteriocins (<5 kDa) consist of small membrane-active, proteolysis- and heat-resistant peptides made of 19–50 amino acids. These bacteriocins are post-translationally modified resulting in the nonstandard amino acids, such as lanthionine, dehydroalanine β-methyllanthionine, labyrinthine, and dehydrobutyrine. Also, class I is subdivided into class Ia (lantibiotics), class Ib (labyrinthopeptins), and class Ic (sanctibiotics) [16].

Class II bacteriocins (<10 kDa) are made up of four subtypes (two-peptides, pediocin-like, circular, and nonpediocin-like linear). They are heat-stable, pH-resistant, nonmodified small peptides. Class II bacteriocins are subdivided into class IIa (pediocin-like bacteriocins), class IIb (two-peptides unmodified bacteriocins), class IIc (circular bacteriocins), and class IId (unmodified, linear, nonpediocin-like bacteriocins) [17].

Pediocin-like bacteriocins are the most prevalent class IIa bacteriocins [17], whereas bactofencin A is a class IId bacteriocin very similar to eukaryotic cationic antimicrobial peptides [18].

Class III bacteriocins, also known as bacteriolysins, incorporate large, heat-labile proteins (with a molecular weight higher than 10 kDa). Based on their mode of action, they can be classified as class IIIa or bacteriolysins (lytic bacteriocins) and class IIIb or nonlytic bacteriocins, which affect the cell membrane potential [19].

This class of bacteriocins is comprised of lactacin A and B, lysostaphin (staphylococcin bacteriocin), acidophilus A, helveticin V-1829, and helveticin J, as well as bacteriocins produced by Gram-negative bacteria (pyocins and salmocins produced by *Pseudomonas* and *Salmonella* species) [20].

Class IV bacteriocins are complex proteins that depend upon essential lipid or carbohydrate conjugation in order to be active [17]. However, some reports classify these protein-macromolecule complexes as hydrolytic polypeptides and not as bacteriocins [21].

## 3. Bacteriocins to the Rescue in Microbial Infections

Generally, bacteriocins are active against species phylogenetically related to the bacteriocin-producing bacteria itself (narrow spectrum) [25] or across genera (broad spectrum).

Bacteriocins can inhibit growth of pathogens in order to defend their producer by acting as pore-forming agents or by causing membrane perturbations [26].

**Figure 2:**
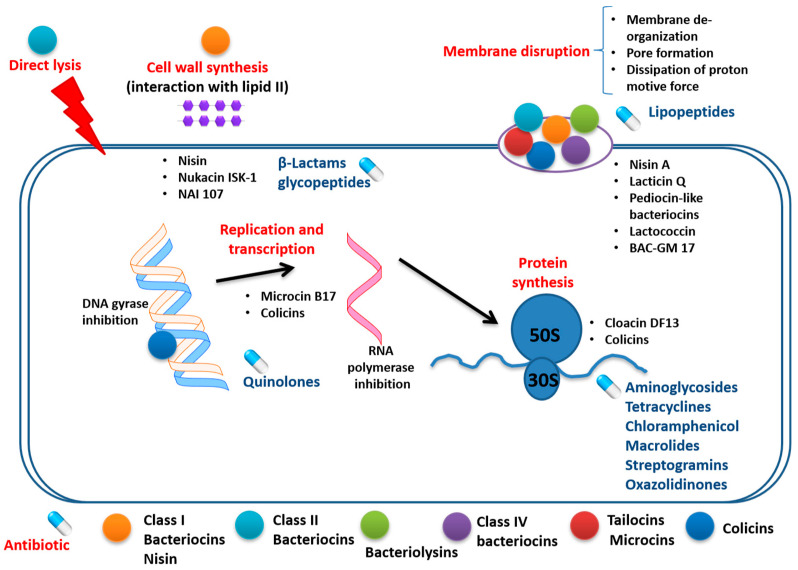
Common targets for antibiotics and bacteriocins; CC-BY-4.0 license.

Several differences exist between bacteriocins and antibiotics and these include: (1) mode of synthesis (while antibiotics are secondary metabolites, bacteriocins are synthesized on the bacterial ribosomal surface; (2) mechanisms of action that differ from those of antibiotics that can be divided into those that function primarily at the cell envelope and those that act primarily within the cell, affecting gene expression and protein production; and (3) generally, bacteriocins are less temperature-labile compared to antibiotics and can withstand extreme pH. Their stability is due to their complex structure characterized by various post-translational modifications (nonconventional amino acids, cyclization, disulphide bridges). Unlike antibiotics, bacteriocins may be sensitized to proteases because of their peptide backbone [16]. Both bacteriocins and antibiotics can affect various processes in the target cell such as cell wall synthesis, membrane integrity, nucleic acid replication and translation, and protein synthesis. The comparative mechanisms of action characteristic for various bacteriocin classes and antibiotics are shown in Figure 2.

Bacteriocins also act as signaling peptides. They can signal other bacteria through bacterial cross talk and quorum sensing within microbial communities or send signals to cells of the host immune system [27]. In addition, they can enhance the beneficial effects of probiotics and may even exhibit antiviral and anticancer activity [26,28,29].

## 4. Bacteriocins Produced by Gram-Positive Bacteria

Nisin A, the most common class I bacteriocin, which has a generally regarded as safe (GRAS) status and is approved by Food and Drug Administration (FDA) as food additive since 1988, is ribosomally produced by *Lactococcus lactis* strains. Nisin A has a complex mechanism of action: inhibition of cell wall synthesis via masking of lipid II (bacteriostatic) as well as membrane insertion leading to pore formation (bactericidal). Nisin A affects numerous Gram-positive genera including *Staphylococcus, Listeria, Streptococcus, Clostridium difficile, Bacillus,* and *Enterococcus* [30]. Bacteriocins are mainly active against Gram-positive bacteria and less effective on Gram-negatives, mainly due to the outer membrane, which hinders the access to its target, lipid II. Nevertheless, reports show that nisin combined with antibiotics can have effects against Gram-negative pathogens [31]. A recent study highlighted the efficiency of nisin in combination with polymyxin in combating *P. aeruginosa* biofilms [32]. It was shown that, in the presence of nisin, the amount of polymyxin required to disrupt *P. aeruginosa* biofilms was significantly lower. It is possible that polymyxin may facilitate the transit of nisin to its target [33]. In addition, the synergistic activity of nisin with clarithromycin against *P. aeruginosa* and other non–β-lactam antibiotics against strains of vancomycin-resistant enterococci and MRSA was reported [34,35]. In addition to its antibacterial effects, nisin also affects fungi (i.e., *Candida albicans*) and decreases tumorigenesis both in cell lines and animal models [36]. Nisin A and its variants are the main members of lantibiotics with other members of this class consisting of lactosin S, lacticin 481, carnocin U149, and the *Bacillus* peptides subtilin and subtilosin A [30]. Recently, O’Sullivan et al. described nisin J, a natural nisin generated by a staphylococcal human skin isolate [37].

Several reports show that lantibiotics with various modes of action can be used to counteract MRSA biofilms, having the potential to prevent or cure biofilm-associated infections, such as nukacin ISK-1, a lantibiotic generated by *Staphylococcus warneri* ISK-1 [38,39].

Mersacidin produced by *Bacillus* species was demonstrated to act on *S. aureus* cell wall at very low concentrations and current studies are focused on the development of a mutant peptide of mersacidin with improved antimicrobial activity as a potential therapeutic agent that could be effective against antibiotic-resistant bacteria [40].

Lacticin Q, a class Id bacteriocin generated by *Lactococcus lactis* QU 5, has a bactericidal mode of action by forming toroidal pores that cause protein leakage from target cells and it was shown to be effective against MRSA biofilms [39].

The screening by O’Sullivan et al. (2019) reported the isolation of 13 novel bacteriocin-producing human skin isolates that could be useful as probiotics for topical skin applications in order to restore the normal microbiota through their inhibitory activity against skin pathogens such as MRSA and *Cutibacterium acnes* [41].

In a recent study by Ansari et al. (2018), a pH- and temperature-stable bacteriocin from *Bacillus subtilis* KIBGE-IB17 (BAC-IB17) was shown to be efficient against MRSA strains [42].

Purified bacteriocins from *Lactobacillus*, *Enterococcus, and Pediococcus genera* alone or in combination with antibiotics (tigecycline, polymyxin B, imipenem, and cefotaxime) showed increased activity against MDR (Multidrug-resistant) clinical pathogens *E. coli* (GN9, IB9, GN13), harboring *bla*_CTX-M_, *bla*_SHV,_ and *bla*_NDM,_ and *K. pneumoniae* KP7 [43]. *Enterococcus mundtii* was reported to produce ST4SA, a class Iia peptide with activity against *P. aeruginosa*, *S. aureus*, *S. pneumoniae*, *E. faecium, E. faecalis,* and *Acinetobacter. baumannii* [44].

## 5. Bacteriocins Produced by Gram-Negative Bacteria

The first description of bacteriocin-mediated inhibition was reported in 1925 in antagonistic isolates of *E. coli*. Based on their molecular mass, they were classified into colicin-like bacteriocins (30–80 kDa) that specifically target *E. coli* and microcins (1–10 kDa). Colicins are mainly located in plasmids with few chromosomally encoded. These large proteins consist of three domains: an amino-terminal domain that mediates the target cell outer-membrane transport, a receptor-binding domain that mediates the transport into the periplasm, and a carboxy-terminal cytotoxic domain that exhibits the inhibitory effect. There are three main mechanisms of actions described for colicins: nuclease activity, i.e., DNA/RNA hydrolysis of the target cell, formation of pores that impairs the membrane integrity, and inhibition of the murein synthesis. An immunity protein is produced by the colicin-like producer strain in order to defend them from its own bacteriocin.

Microcins are a group of potent antibacterial peptides exhibiting a diversity of structures that combine the self-immunity, leader peptides, and maturation steps of bacteriocins from Gram-positive bacteria with the uptake mechanism of colicins. Their mode of action is comparable to that of a “Trojan horse”: The outer membrane receptors of susceptible bacteria recognize them as siderophores, but intracellularly, they target enzymes with role in DNA/RNA structure or synthesis, i.e., DNA gyrase GyrB (MccB17) inhibits RNA polymerase (MccJ25) or the ATP synthase (MccH47). In contrast to colicins, they do not affect peptidoglycan synthesis [22]. Microcins can be considered as future potent antibacterial agents [23]. Natural microcin J25 (MccJ25) may be a potential alternative to traditional antibiotics for the management of antibiotic-resistant infections. Studies suggest that recombinant MccJ25 may be an efficient alternative for prevention and treatment of *E. coli* and *Salmonella* infections, being used in the food industry or in veterinary and agriculture applications [24].

Colicins were demonstrated to be able to control multidrug-resistant *E. coli* serotype O104:H4 (strain ATCC^®^ BAA-2326TM) [45], antibiotic-resistant *E. coli*, and Shiga toxin-producing *E. coli* [46]. In vitro studies have highlighted the role of bacteriocins against biofilm-embedded and planktonic bacteria. Interestingly, it was shown that colicin R preferentially targets bacteria embedded in biofilms [47,48].

Pseudomonads produce an armamentarium of bacteriocins that varies from strain to strain. Four groups have been so far identified: lectin-like bacteriocins, modular bacteriocins, tailocins, and B-type microcins. Self-inhibition as a result of toxin activity in bacteriocin-producing strains is managed by co-expression of specific immunity genes. Tailocins, also known as high-molecular-mass bacteriocins, have a structure similar to the tail structures of bacteriophages from the Siphoviridae and Myoviridae families [49]. The F-type and R-type pyocins of *P. aeruginosa*, exhibiting morphological similarities to P2-like temperate enterophages and λ phage, respectively, are the best studied. Modified R-type tailocins were shown to eradicate *P. aeruginosa* acute peritonitis in an infection model [50].

Lectin-like bacteriocins (LlpAs) share structural similarity with plant lectins and are organized in two B-lectin domains and a short carboxy-terminal chain [51]. Their mechanism of killing is not completely elucidated.

**Figure 3:**
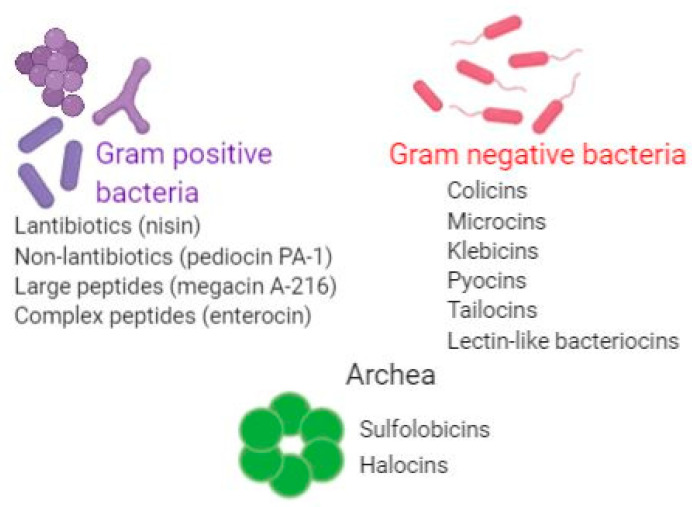
Main examples of bacteriocins produces by Gram-positive and Gram-negative bacteria as well as those produced by Archaea (original figure, made using biorender.com).

Figure 3 summarizes the main types of bacteriocins produced by Gram-positive and Gram-negative bacteria.

## 6. Bacteriocins Produced by Archaea

Studies have also reported archaeal members as bacteriocin producers. Archaeocins such as sulfolobicins and halocins have been described [52] (Figure 3). Halocins, produced by halobacteria, are produced during late exponential to early stationary growth phase and they target the cell membrane by inhibiting the Na^+^/H^+^ antiporter and proton flux or by changing cell permeability [53]. This leads to cell swelling and further lysis. Other known archaerocins are sulfolobicins, which are produced by *Sulfolobus islandicus*. Sulfolobicins are intracellular and membrane-associated narrow spectrum bacteriocins that counteract the growth of closely related strains. However, their mechanism of action still needs to be deciphered [54].

## 7. In Vivo Activity of Bacteriocins

Several studies have addressed the in vivo effects of bacteriocins. For instance, the lantibiotic NAI-107, lacticin 3147, and nisin exhibited bactericidal activity against MRSA and VRE in animal models [55,56]. Bacteriocin Abp118 produced by *Lactobacillus salivarius* UCC118 successfully colonized in mice challenged with *L. monocytogenes* [57]. Sublancin was shown to prevent MRSA-related intraperitoneal infection in mice, as revealed by the significantly reduced mortality rates and weight loss of MRSA-challenged animals [58]. Nisin F produced by *L. lactis* F10 given intranasally proved to be efficient for the treatment of respiratory infections rats artificially infected with *S. aureus* regardless of their immune status [59]. In a lethal peritonitis murine model, R-type pyocin prevented death from 90% lethal dose inocula of a pyocin-sensitive *P. aeruginosa* clinical isolate [60].

Using a model of mouse gut colonization with *E. faecalis* and the conjugative plasmid pPD1-expressing bacteriocin 214, Kommineni et al. showed that bacteriocin expression by commensal bacteria can influence niche competition in the GI (gastrointestinal) tract. It was suggested that bacteriocins, delivered by commensals from a precise intestinal niche, may specifically eliminate intestinal colonization by multidrug-resistant bacteria, without causing microbiome disruption [61].

Modified R-type bacteriocins, or Avidocin-CDs, were developed as alternative agents that specifically kill *C. difficile* strains. Preclinical animal studies indicated that these molecules could potentially be employed as prophylactic agents to prevent *C. difficile* infections. Importantly, since these agents maintain the indigenous microbiome unaltered, they could be safe for administration as prophylactic agents without making patients susceptible to enteric infection after the treatment [62].

Bioengineering has been successfully applied for various bacteriocins. For instance, the use of codon optimization improved the yield of Enterocin A, a class IIa bacteriocin secreted by *E. faecium* CTC492 229 [63]. Mutated peptides with residues replaced within the N-terminus of pediocin PA-1 were reported to be efficient against *S. aureus* [64]. Conversely, the variants within the C-terminus harbored increased activity against *L. monocytogenes* [65]. In addition, bioengineered S29A and S29G nisin variants harbored improved activity against Gram-negative bacteria [28,31]. A hybrid bacteriocin (Ent35–MccV), resulting from the fusion of the microcin V and enterocin CRL35 genes (*munA* and *cvaC*), had activity against *Listeria monocytogenes*, S*. epidermidis*, *E. coli*, *Serratia* *marcescens*, and *K. pneumoniae* [66].

## 8. Challenges and Opportunities

The high diversity and relative abundance of bacteriocins favor their use as alternative therapeutics in the infectious disease management. These potent antimicrobials have been extensively studied in the last decade and resulted in different applications such as food preservation, medical treatments, and personal care. A major advantage in the development of diverse applications is the fact that they are recognized as GRAS substances by the United States Food and Drug Administration (FDA) and the European legislation regarding pharmaceutical and food industry uses.

Bacteriocins may be employed as potential candidates to take the place of antibiotics as active agents against antimicrobial-resistant pathogens. Besides the emergence of resistance, conventional antibiotics trigger microbiota imbalances (dysbiosis) induced by broad-range killing of bacteria [47]. Unlike antibiotics, most bacteriocins hold a narrow spectrum of activity. This means that the bacterium responsible for the infection needs to be identified prior to treatment and, consequently, one species will be targeted for killing, leaving the rest of microbiota intact. Moreover, the narrow killing spectrum will reduce the selective resistance pressure on bystander microbes [67]. However, while emergence of resistance to conventional antibiotics is well known, we have scarce information about how bacteriocin resistance may appear and, more importantly, how it will evolve in vivo. Several studies suggest that resistance to bacteriocins may occur via modifications of cell surface receptors, depending on environmental factors [67,68].

It is of paramount importance to address the issue of emergence of resistance when bacteriocin-based antimicrobial strategies are proposed for clinical use. So far, our understanding of the potential for bacteriocin resistance development comes primarily from in vitro studies [69].

Generally, resistance mechanisms to antimicrobial peptides include: (1) enzymatic inactivation by peptidases (elastase, metalloprotease), as described in *P. aeruginosa*, *Burkholderia cenocepacia, E. faecalis*, Group A *Streptococcus, Proteus mirabilis, S. aureus*, *E. coli* pathovars, *S. enterica* serovar *typhimurium, Bacillus anthracis*, *B. subtilis,* and *Porphyromonas gingivalis;* (2) changes in the antimicrobial peptide target (*S. aureus, Mycobacterium marinum*, Group A *Streptococcus*); (3) cellular filamentation; (4) entrapment by secreted molecules that can bind and neutralize antimicrobial peptides; (5) impermeability due to changes in cellular surfaces; (6) chemical modifications of the Gram-negative lipopolysaccharide lipid A (*V. cholerae* O1El Tor, *Salmonella* sp., *Burckholderia caepacia*; *E. coli, Helicobacter pylori, Yersinia enterocolitica;* (7) D-alanylation of teichoic acids in Gram-positive bacteria to diminish the negative charges in their surface; (8) capsule synthesis to avoid contact between the microbial surface and cationic antimicrobial peptides (*K. pneumoniae*, *P. aeruginosa, Streptococcus pneumoniae* serotype 3, *Neisseria meningitidis,* and *Campylobacter jejuni*); and (9) efflux pumps (*Neisseria gonorrhoeae*, *S. enterica*, *K. pneumoniae*, *H. influenzae*, *Y. enterocolitica*, *S. aureus, S. pneumoniae*, *C. albicans* [70].

Bacteriocin resistance has been documented for nisin, lysostaphin, lacticin 3147, and pediocin-like bacteriocins, [71]. Several mechanisms involved in bacteriocins’ resistance have been described. Immune mimicry has been described as a mechanism ensuring protection specifically against bacteriocins. Thus, non-bacteriocin-producing strains harbor what is called “orphan immunity genes” by encoding functional homologues of bacteriocin immunity systems. This trait has been reported for class II bacteriocins and lantibiotics [56,72]. Resistance may arise also due to bacteriocin degradation. For example, several nisin-resistant strains of *Bacillus *spp. secrete nisinase, an enzyme breaking the C-terminal lanthionine ring of nisin [73].

However, even if resistance occurs, many bacteria still remain sensitive to a certain bacteriocin level. Unlike other known therapeutic compounds, some bacteriocins (i.e., lantibiotics) possess a dual mechanism of action, a fact that lowers the probability of selecting resistant strains. Nevertheless, careful consideration must be taken if and when bacteriocins will be used clinically to overcome loss of efficacy and spread of resistance.

While some antibiotics trigger damaging collateral effects on host health, bacteriocins were shown to have low or no cytotoxicity [74]. The lack of toxicity is a result of the fact that the healthy human gastrointestinal tract is highly colonized by bacteriocin-producing commensal strains and the bacteriocins originating from lactic acid bacteria have long been used in fermentation products as biopreservatives.

Several bacteriocins (class II bacteriocins, nisin, other lantipeptides) were reported to be noncytotoxic on different eukaryotic cell lines even when used at very high doses [75,76]. While bacteriocins clearly exhibit features beneficial in treating infectious diseases, one must highlight the fact that several Gram-positive bacteria may use bacteriocins as potential virulence factor for higher pathogenicity. For instance, the lantibiotic cytolysin of enterococcal origin was shown to be cytotoxic against a wide array of cell lines, including human intestinal epithelial cells, horse red blood cells, retinal cells, and polymorphonuclear leukocytes [74]. Furthermore, pathogenic streptococcal strains secrete bacteriocin virulence factors including streptolysin S and hemolysins’ intermedilysin, which are involved in invasive *Streptococcus* group A infection [77]. Microcin E492 [78] was also reported as cytotoxic. Hence, cytotoxicity needs to be addressed for each one of the bacteriocins aimed for human use. Moreover, cytotoxic bacteriocins may serve as antitumoral agents considering they usually are inserted into the negatively charged membranes of cancer cells [79]. Few preliminary in vivo data regarding the cytotoxic effect of bacteriocins on the host or its immune response are available. Most studies report no adverse effects against the host organism. However, Bird and Grieble [80] reported an 11% mortality rate in pyocin-treated chick embryos, with the control group having a 6% mortality rate from injection alone: In this case, it was unclear whether the pyocin preparation used was free of endotoxin. Subsequently, more research is needed regarding the dosing and administration timing of bacteriocin during the course of infection. To solve this challenge, robust pharmacokinetic studies and optimized infection models are needed.

Due to their small size, non-immunogenic nature, biocompatibility, and biodegradability, bacteriocins are a promising replacement for antibiotics. However, for their potential implementation for medical use, several issues need to be addressed, including solubility, stability at different pH values, purification, and large-scale production. Bacteriocins exhibit a complex molecular structure comprised of post-translational modification, which would be costly to reproduce on a large scale [16].

Route of administration is another aspect that needs to be carefully chosen and optimized. The conditions in the human gut are highly variable, in terms of food particles’ size, digestive enzymes, salts, spices, bile, etc., all of which trigger changes in bacteriocin production. Thus, oral administration of bacteriocins needs to tackle many variables to be considered in terms of bacteriocin activity in the gastrointestinal tract half-life, intestinal absorption and bioavailability, pH stability, interaction with food particles and with other microbes in the gut, resistance to digestive enzymes, and renal clearance.

This can be addressed by employing alternative routes such as intravenous, topical, or intranasal administration. Bacteriocins can be administered via the parenteral route, in case of systemic infections, but in this case, they can be inactivated by bloodstream proteases (such as those involved in fibrinolysis or hemostasis) and this may reduce their activity. Since they are sensitive to proteases in vivo, bacteriocin peptides may display lower half-life compared to antibiotics [16]. In light of this, further studies are needed to analyze peptide modification of bacteriocins to provide structural information to remove the recognition sites of proteases.

However, due to their reduced half-lives and lack of specificity, the current administration techniques of antimicrobial peptides need high doses, leading to emergence of associated side effects. Hence, targeted delivery using adequate carriers is a necessity [81,82]. The advent of nanotechnology has enabled the development of novel approaches for delivery of antimicrobial peptides. Nanodelivery systems comprised of different nanoparticles (i.e., polymer, lipid, carbohydrate, or metal based) can be exploited to efficiently target these antimicrobial peptides in the infected host. Unlike free bacteriocins, nano-formulated bacteriocins were reported to have broader spectrum of antimicrobial activity and higher stability [82]. In the food sector, nano-encapsulation of bacteriocins ensures protection against degradation by proteolytic enzymes, making them more stable and improving their activity against food-spoiling microorganisms [83].

Liposomes are nontoxic, biodegradable spherical structures made of phospholipid bilayer membranes surrounding an aqueous medium [84], which have been extensively used to encapsulate various bioactive compounds, including bacteriocins [85,86]. For instance, nisin Z was successfully encapsulated in nanoliposomes prepared from nanoliposomes composed of dipalmitoyl phosphatidylcholine/dicetylphosphate/cholesterol with a 7:2:1 molar ratio [87]. Moreover, pediocin AcH was successfully loaded into phosphatidylcholine nanovesicles with high stability, high entrapment efficiency (80%), and antimicrobial activity [88]. However, while liposome-encapsulated pediocin maintained its antimicrobial activity for a longer period, this activity was reduced compared to that of free pediocin [88].

Solid lipid nanoparticles (SLN) have a solid triglyceride core, which makes them suitable for slow drug-release formulations [89]. SLN can protect bacteriocins against degradation, extending their antibacterial activity for longer periods of time. Unlike free nisin, SLN containing nisin exhibited significantly longer activity against *L. plantarum* TISTR 850 (for up to 15 days) and *L. monocytogenes* DMST 2871 (for 20 days) [89,90].

Chitosan nanoparticles have also been used for bacteriocin delivery. For example, chitosan combined with alginate was used for the encapsulation of nisin, with a 95% entrapment efficiency [91]. Moreover, nisin-loaded chitosan/alginate expressed a much higher level of activity against *S. aureus* ATCC 19,117 and *L. monocytogenes* compared to the antimicrobial activity of free nisin [91,92].

Recent studies highlight the use of nanofibers as delivery systems. Thus, bacteriocins as well as other beneficial substances can be electrospun into nanofibers to act against multidrug-resistant nosocomial pathogens. Heunis et al. reported that an antimicrobial nanofiber wound dressing containing nisin electrospun into equimolar amounts of poly (D, L-lactide) (PDLLA) and poly (ethylene oxide) (PEO) was effective against *Streptococcus* and *Staphylococcus* [93]. Ahire et al. investigated the activity of nisin incorporated into PEO-PDLLA and 2,3-dihydroxybenzoic acid (DHBA) nanofibers [94]. This nanoformulation showed activity against MRSA biofilms [94]. Since iron is required in the process of biofilm formation and DHBA has the ability to chelate iron, it is not surprising that biofilm formation decreased by 88% 24 h after the exposure to nanofibers containing DHBA and nisin [94]. In addition, co-incorporation of silver nanoparticles and nisin into nanofibers led to enhanced antimicrobial activity against a wide array of pathogenic bacteria [94].

Despite the plethora of advantages that these delivery systems offer, they still have some limitations. Each of these delivery approaches has its own challenges, which need to be addressed to ensure practicality of the approach. A full analysis of the physiological, physicochemical, and molecular processes triggered by these delivery systems needs to be taken into consideration. More studies are needed to assess whether the use of these nanodelivery systems enhance the antimicrobial properties of bacteriocins. Moreover, the interactions between these peptides and nanomaterials and the targeted microbes need to be characterized. Even though preliminary studies (mostly in vitro) hold promise, human testing comes with its own hurdles (i.e., bioaccumulation, biokinetics, and toxicity issues). Thus, pharmacokinetic profiles’ clinical translational studies, including long-term toxicity and pharmacokinetic profiles, should be performed to address fundamental issues in terms of their clinical feasibility.

Even though they are a potential tool to curtail infections, there is a paucity of clinical trials using bacteriocins. So far, NVB302, a derivative of the lantibiotic deoxyactagardine B has been used in a clinical trial for *C. difficile* infection treatment [95]. Peptide IB-367 has recently undergone phase I safety trials on humans for use against chronic *P. aeruginosa* lung infections, specifically on cystic fibrosis patients.

Nisin and IB-367, a protegrin-like cationic peptide produced by Intrabiotics (Mountain View, CA, USA), have reached phase I clinical trials for acne treatment, whereas nisin A and Z are in preclinical trials for combating vancomycin-resistant enterococci. Nisin was also used in a recent clinical trial to assess its inhibitory effects on pathogens associated with ventilator-associated pneumonia (*P. aeruginosa, A. baumannii, S. aureus,* and *K. pneumoniae*).

Even though these clinical studies reveal a positive impact of bacteriocins in overcoming various infections, research is still needed in this field.

## 9. Concluding Remarks

So far, the development of new antibiotics is not fast enough to manage microbial infections. In this scenario, therapeutic alternatives are urgently needed. Undoubtedly, bacteriocins may play a significant role in fighting antibiotic-resistant bacteria due to their narrow-target activity, low toxicity, and high stability and specificity. Several bacteriocins, notably nisin, were shown to harbor activity against Gram-negative species, sporicidal activity as well as anti-biofilm activity, further highlighting their importance in infectious disease management. Importantly, the function of bacteriocins in probiotics is a complex one and not fully understood. Therefore, further studies should be performed in order to address their in vivo effects, mechanism of action, the impact with the host immune system and the microbiota, and large-scale production costs as well as the emergence of bacteriocin resistance.

## Figures and Tables

**Table 1:** Bacteriocin classes’ characteristics, spectrum, and mode of action.

Class	Subclass	Examples	Characteristics	Antimicrobial Spectrum	Mechanisms of Action	References
I	Ia (lantibiotics)	Nisin, lacticin 481, lactosin S, carnocin U149, subtilin subtilosin A Mersacidin	small membrane-active, proteolysis- and heat-resistant peptides (<5 kDa)	MRSA, *Listeria* spp., *Streptococcus* sp. *Clostridium difficile, Bacillus, Enterococcus, C. albicans*	Pore formation Cell wall synthesis	[16]
	Ib(labyrinthopeptins)					
	Ic (sanctibiotics)					
II	IIa (pediocin-like bacteriocins), IIb (two-peptides unmodified bacteriocins), IIc (circular bacteriocins) IId (unmodified, linear, nonpediocin-like bacteriocins)	Pediocin PA-I, pediocin AcH, enterocin A Uberolysin, carnocyclin, circularin A and AS-48, Grassericin A/reutericin A	heat-stable, pH- resistant, nonmodified, small peptides (<10 kDa)	*E. coli, Listeria monocytogenes*, *Staphylococcus epidermidis*, *Serratia marcescens*, *K. pneumoniae,* *MRSA*	Pore formation	[14,15,16,17]
III	**Gram positive:** lysostaphin, lactacin A and B helveticin V-1829, helveticin J, helveticin M acidophilus A		large heat-labile proteins (with a molecular weight higher than 10 kDa	*S. aureus, S. saprophyticus, Enterobacter cloacae* *Gardnerella vaginalis*, *Streptococcus agalactiae*, *P. aeruginosa*	Pore formation	[15,16,19,20]
	**Gram negative:** pyocin salmocins			*P. aeruginosa* *Salmonella* sp. *STEC*	Pore formation	[22,23,24]

## Data Availability

Not applicable.
